# ALAD-YOLO:an lightweight and accurate detector for apple leaf diseases

**DOI:** 10.3389/fpls.2023.1204569

**Published:** 2023-08-17

**Authors:** Weishi Xu, Runjie Wang

**Affiliations:** ^1^ School of Intelligent Science and Technology, East China University of Science and Technology, Shanghai, China; ^2^ School of Geological Engineering, Tongji University, Shanghai, China

**Keywords:** apple leaf disease, lightweight object detection, ALAD-YOLO, coordinate attention, group convolution

## Abstract

Suffering from various apple leaf diseases, timely preventive measures are necessary to take. Currently, manual disease discrimination has high workloads, while automated disease detection algorithms face the trade-off between detection accuracy and speed. Therefore, an accurate and lightweight model for apple leaf disease detection based on YOLO-V5s (ALAD-YOLO) is proposed in this paper. An apple leaf disease detection dataset is collected, containing 2,748 images of diseased apple leaves under a complex environment, such as from different shooting angles, during different spans of the day, and under different weather conditions. Moreover, various data augmentation algorithms are applied to improve the model generalization. The model size is compressed by introducing the Mobilenet-V3s basic block, which integrates the coordinate attention (CA) mechanism in the backbone network and replacing the ordinary convolution with group convolution in the Spatial Pyramid Pooling Cross Stage Partial Conv (SPPCSPC) module, depth-wise convolution, and Ghost module in the C3 module in the neck network, while maintaining a high detection accuracy. Experimental results show that ALAD-YOLO balances detection speed and accuracy well, achieving an accuracy of 90.2% (an improvement of 7.9% compared with yolov5s) on the test set and reducing the floating point of operations (FLOPs) to 6.1 G (a decrease of 9.7 G compared with yolov5s). In summary, this paper provides an accurate and efficient detection method for apple leaf disease detection and other related fields.

## 1 Introduction

Apple is one of the most important crops with rich nutritional and medicinal values and is widely grown in the world. China, one of the largest apple producers, produced more than 41 million tons of apples in 2019, accounting for 54.07% of the global total (Hu et al., 2022). However, apples are often threatened by various foliar diseases caused by bacterium, such as brown spot disease and mosaic disease, which will lead to a drastic decrease in apple yield and quality without timely detection and prevention, causing significant economic losses to farmers. Therefore, an efficient and accurate diagnosis method for apple foliar diseases is essential to promoting the development of the apple industry, which is of great practical value.

Traditionally, apple leaf diseases are usually detected by manual inspection, which has many limitations and drawbacks. First, the method relies on professional inspectors for detection (Liu et al., 2017), thus making the limitation of human resources a serious problem. Secondly, the accuracy of the method is difficult to guarantee due to factors such as the vision and fatigue of human eyes (Dutot et al., 2013). Especially for large-scale planting areas such as apple plantations, the use of manual detection methods will lead to a large workload, which will not only be labor-intensive but also cause missed or false detection. Therefore, automated apple leaf disease detection has become a hotspot for research (Zhang and Tao, 2021) to improve the efficiency and accuracy of detection.

With the development of computer science and technology, machine learning algorithms have been applied to the agricultural field. For example (Pallathadka et al., 2022), preprocessed the images with histogram equalization, then applied the principal component analysis algorithm for feature extraction, and finally used the support vector machine and naïve Bayes to classify rice leaf diseases. However, machine learning algorithms are usually constrained by the ultra-high computational effort during the stages of data preprocessing and feature extracting, making the utility of these methods generally poor (Sujatha et al., 2021). compared performances of machine learning and deep learning algorithms for plant leaf disease detection, and the experimental results show that the latter has better performance on this kind of task.

With the rise of convolutional neural networks and the creation of residual structures, deep learning techniques have achieved a technological leap in a very short period of time, which also improves the performances of target detection algorithms with their excellent feature extraction and model migration ability. Target detection algorithms have evolved from two-stage detection algorithms to one-stage detection algorithms in this phase. Among them, two-stage detection algorithms such as Faster R-CNN (Ren et al., 2015) and Mask-RCNN have been applied to detect plant leaf diseases by many scholars, such as (Li et al., 2023) combined Mask-RCNN with the geometric model to improve the ground-penetrating radar (GPR), and the pixel-level segmentation for localization can effectively improve the detection accuracy, reaching an average depth prediction error of 2.78 cm (Du et al., 2022). proposed a corn pest detection algorithm based on faster R-CNN, called pest R-CNN, which classified pest invasion severity into four categories, juvenile, mild, moderate, and severe based on feeding severity, and determined the severity of infestation and specific forage location. One-stage detection algorithms include YOLO (Tian et al., 2019) and SSD (Jiang et al., 2019), an end-to-end detection method that enables high-speed and real-time detection (LI et al., 2022). constructed the YOLO-JD network by introducing DSCFEM and SPPM modules, increasing the mean average precision (mAP) of jute disease detection to 96.63% (Tian et al., 2019). applied the V-space-based SSD algorithm for multiscale feature fusion and introduced an attention mechanism, increasing the mAP of apple leaf disease detection to 83.19% and the detection speed to 27.53 frames per second (FPS). However, the above models still have the problem of too large model size with a large number of parameters and high computational cost, so lightweight models have become the focus of scholars’ research in recent years (Lin et al., 2023). applied Tiny-YOLOv4 to realize strawberry real-time counting by comparing three frameworks (Darknet, TensorRT, and TensorFlow Lite) and images with different resolutions, allowing the model to achieve an accuracy of 14.6% with 91.95 FPS (Sozzi et al., 2022). proposed an improved Tiny-YOLOX model, called YOLO-Tobacco, for detecting tobacco brown spot disease in an open-air scenario. They incorporated hierarchical mixed-scale (HMU) units and convolutional block attention modules (CBAM), making the detection speed reach 69 FPS but with less accuracy compared with the non-lightweight model.

In summary, scholars have provided a lot of excellent ideas and methods in the field of target detection, achieving good results for plant leaf disease detection. In order to better apply to the actual situation of agricultural production, this paper proposes accurate and lightweight apple detection based on YOLOv5 (ALAD-YOLO), a lightweight network model, taking both detection accuracy and detection speed into account, which can be more easily deployed on mobiles regardless of computational resources. The main contributions are as follows:

(1) We replace the backbone network of YOLOv5 with a more lightweight MobilenetV3s network to reduce the parameter quantity and increase the operational efficiency.
(2) We use the C3 module refined by depth-wise convolution and GhostNet module (DWC3_ghost) to replace all C3 modules in the neck network, improving feature fusion efficiency, reducing computational cost, and maintaining feature expressiveness while minimizing the impact on detection accuracy.
(3) We develop a new module called SPPCSPC_GC, using Cross Stage Partial (CSP) structure, spatial pyramid pooling (SPP) module, and group convolution (GC) to replace the original SPP module at the interface between the backbone network and neck network, making this section better adapted to images of different resolutions, effectively avoiding overfitting, and making the model more lightweight.
(4) We apply the CA mechanism to improve the model’s accuracy. Compared with the CBAM mechanism and the Squeeze-and-Excitation Networks (SE) attention mechanism, the CA mechanism can better capture object spatial and channel information without compromising model lightweighting, thereby improving detection accuracy.

The remaining parts of this article are organized as follows: Section 2 provides a detailed introduction of the dataset and network modules we used. Section 3 presents our experimental results and provides a visual analysis. Next, Section 4 compares and discusses our proposed model with current mainstream networks. Finally, we conclude by summarizing our model and discussing its potential applications.

## 2 Materials and methods

### 2.1 Data collection and preprocessing

#### 2.1.1 Data collection

To improve the generalization ability of the model, the dataset in this paper includes apple leaves with different shooting angles, backgrounds, times, disease ranges, and densities. The selected large amount of image data ensures the model’s ability to detect small disease ranges.

Due to the scarcity of public datasets for apple leaf diseases, this paper collected the Kashmiri Apple Plant Disease Dataset (Kaur et al., 2022) and the public dataset for Plant Pathology 2020-FGVC7 (Raman et al., 2022).

**Figure 1:**
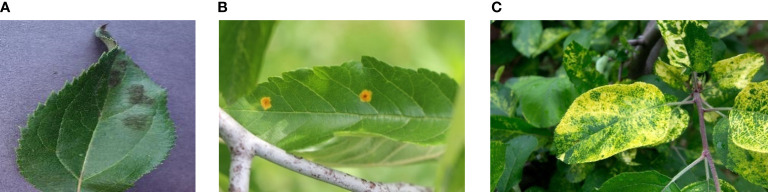
Samples of datasets, where **(A)** is a diseased apple leaf with a normal background, **(B)** is a diseased apple leaf in a real environment, and **(C)** is in an intensive situation where samples of apple leaves have multiple diseases collected in this paper.

However, the above datasets all have problems that do not match the actual detection environment, such as overly clear image backgrounds and mostly displaying single leaves. To enhance the generalization effect of the model and improve its ability to detect small target disease ranges, the dataset also includes 961 small target apple leaf cluster data that we collected ourselves (**Figure 1C**). The final experimental data consists of 2,748 apple disease images.

Most of the images have varying resolutions and large or small detection targets, and they were captured at different angles, providing sufficient overall diversity of the data, as shown in **Figure 1**. The details of the apple leaves are preserved while also being more in line with the actual detection environment; the background is influenced by real outdoor lighting and shadow occlusion. This reflects the actual apple disease leaf detection scene and enhances the robustness and generalization ability of the model training.

LabelMe software was used to generate XML files, and the images were marked as mosaic disease, spot wilt disease, and leaf blight. In the experiment, the dataset was divided into training, validation, and testing sets in an 8:1:1 ratio.

#### 2.1.2 Data preprocessing

**Figure 2:**
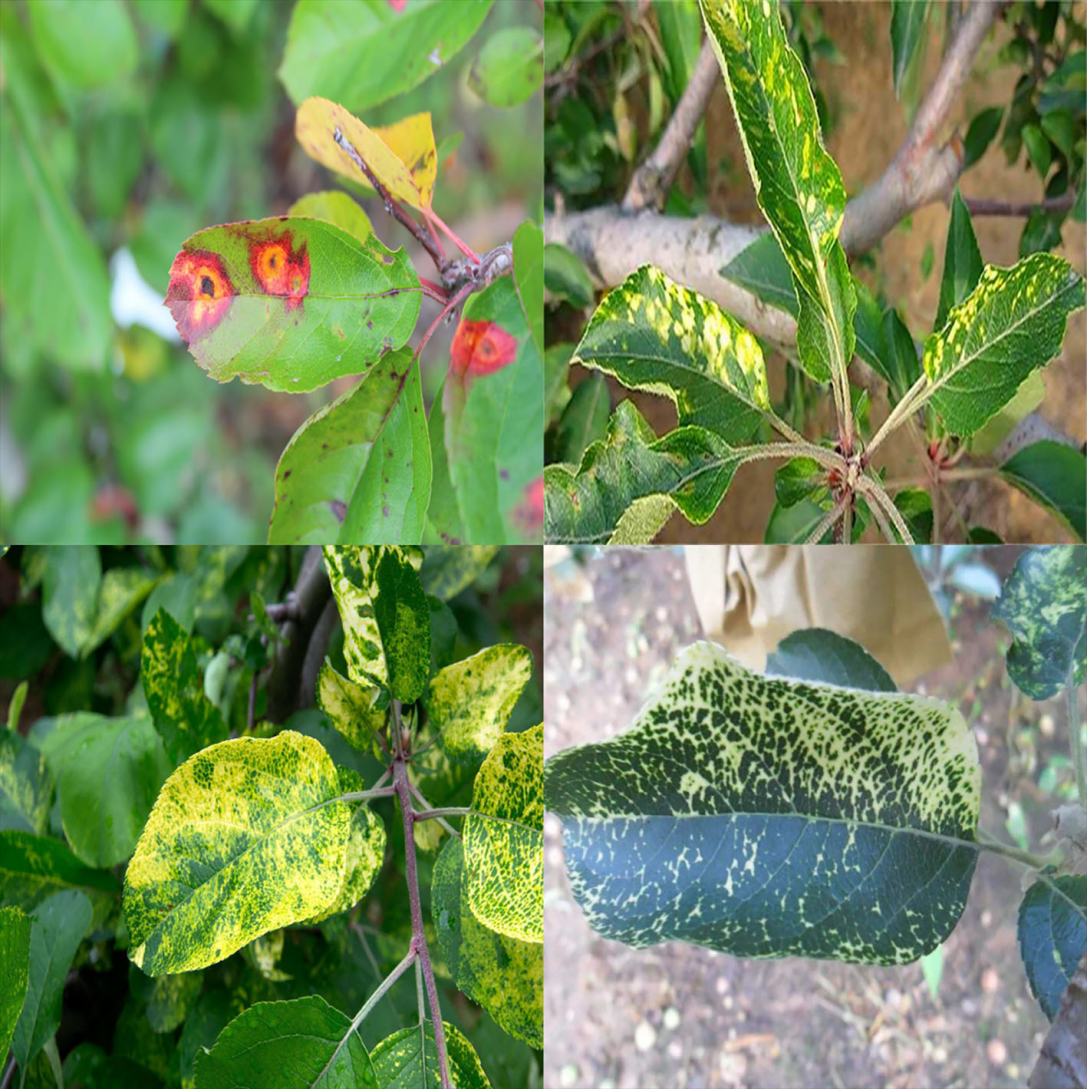
Mosaic data augmentation. Four images are randomly cropped and stitched onto one image as training data.

To improve the generalization ability of object detection models, data augmentation techniques are widely used. We used the mosaic data augmentation technique to preprocess the apple leaf disease detection dataset (Thapa et al., 2020). Mosaic data augmentation is a technique that combines multiple data augmentation operations. It combines four randomly selected images into one and then applies random transformations to the entire image, such as random scaling, flipping, translation, and color change, as shown in **Figure 2**. The probability of scaling and flipping the image is 50%, while the probability of adjusting the hue, saturation, and brightness in color change is 1.5%, 70%, and 40%, respectively (Wang et al., 2021). The probability of translation is 10%.

In the apple leaf disease detection dataset, the distribution of small targets is uneven, which may lead to insufficient model training. By using the mosaic data augmentation technique, we can increase the number of small targets and make their distribution more uniform, thereby improving the model’s detection ability. In addition, mosaic data augmentation can also reduce overfitting and improve the model’s generalization ability.

### 2.2 Design for ALAD-YOLO

**Figure 3:**
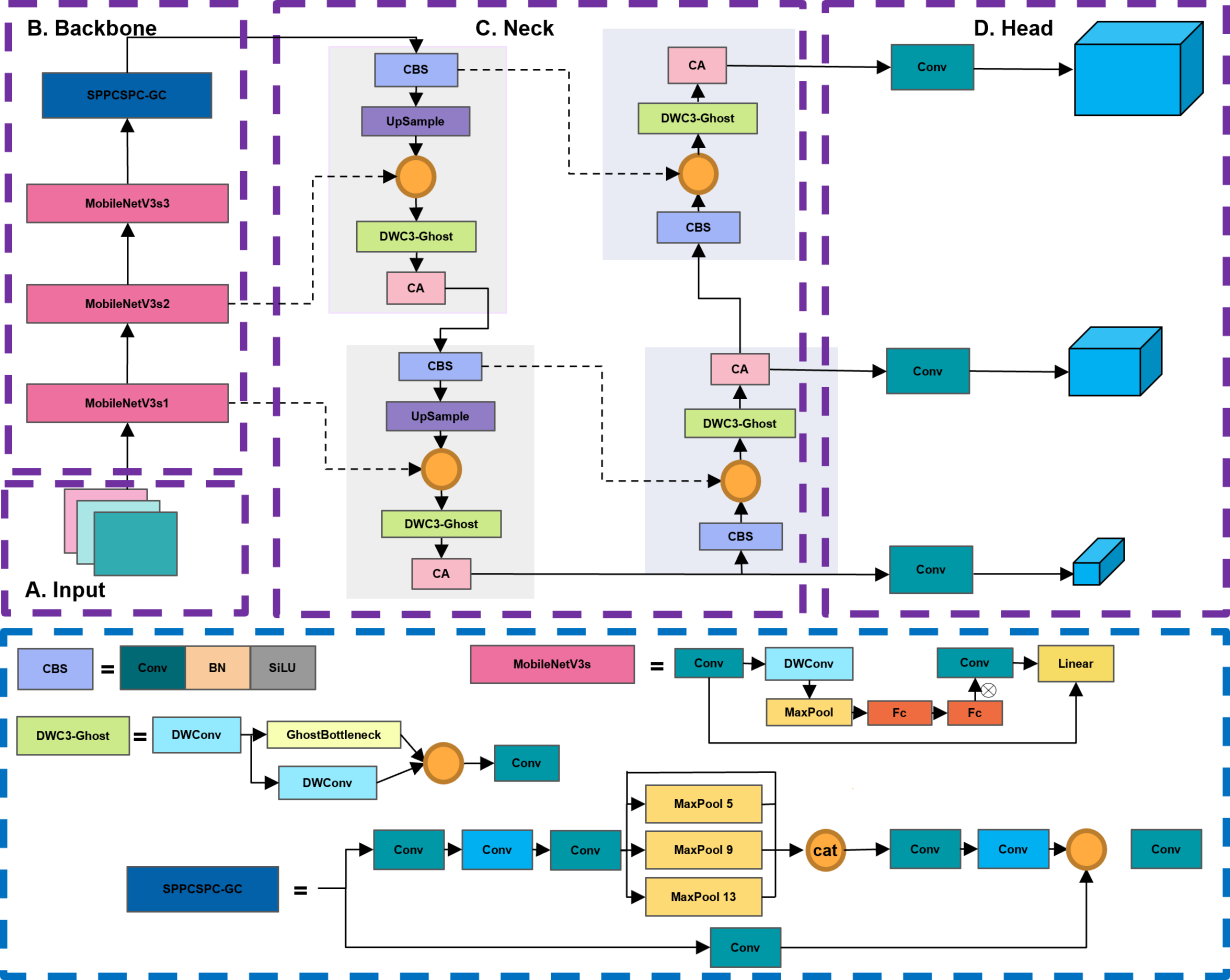
The architecture of the proposed ALAD-YOLO model. The entire network is divided into four parts: input network **(A)**, backbone network **(B)**, neck network **(C)**, and head network **(D)**.

In order to achieve model lightweight and ensure accurate detection of different categories of apple leaf diseases, this paper proposes an efficient and accurate detection network ALAD-YOLO based on YOLOv5s. **Figure 3** shows the detailed structure of the ALAD-YOLO model proposed in this paper.

YOLOv5 and the proposed ALAD-YOLO consist of four main components: the input layer, the backbone network, the neck network, and the prediction head. The basic unit CBS is composed of regular convolution, batch normalization (BN), and activation function SiLU. The backbone network of YOLOv5 is stacked with a large number of CBS modules and C3 modules. The Spatial Pyramid Pooling (SPP) module increases the receptive field of the feature map through three different sizes of pooling kernels, solves the problem of multiscale detection, and connects to the neck network. The prediction head achieves predictions for three different scales of objects and outputs the detection results for large, medium, and small objects.

Due to the large number of CBS and C3 modules in the network, the original YOLOv5 has poor portability (Wadhawan et al., 2012), making it difficult to embed in mobile devices for use in smart agriculture. Therefore, this work proposes an efficient and accurate ALAD-YOLO for detecting apple leaf diseases, as shown in **Figure 3**. The main improvements are as follows: 1) MobileNetV3s basic blocks containing lightweight depth-wise separable convolutions, SE modules, and inverted residual structures are used instead of stacked CBS and C3 modules to improve feature extraction efficiency and compress model size. 2) The DWC3-ghost module is proposed to replace the original C3 module in the neck network to reduce parameter count and FLOPs. 3) The SPPCSPC_GC structure is proposed to replace the original SPP module with group convolution to further compress model size and improve efficiency in the feature fusion stage. 4) A lightweight coordinate attention (CA) module is embedded in the neck network to refine the key information for detecting apple leaf diseases and improve the detection accuracy for different types of diseases.

#### 2.2.1 Lightweight backbone network establishment

The backbone network of YOLOv5 mainly consists of CBS modules and C3 modules, which include convolution operations and residual structures with high parameter count and FLOPs. In order to be applied to embedded mobile devices, while ensuring the detection accuracy of the model, this paper compresses the model parameters as much as possible to improve its portability. A lightweight backbone network for ALAD-YOLO was designed using efficient MobileNetV3 (Howard et al., 2019) building blocks.

**Figure 4:**
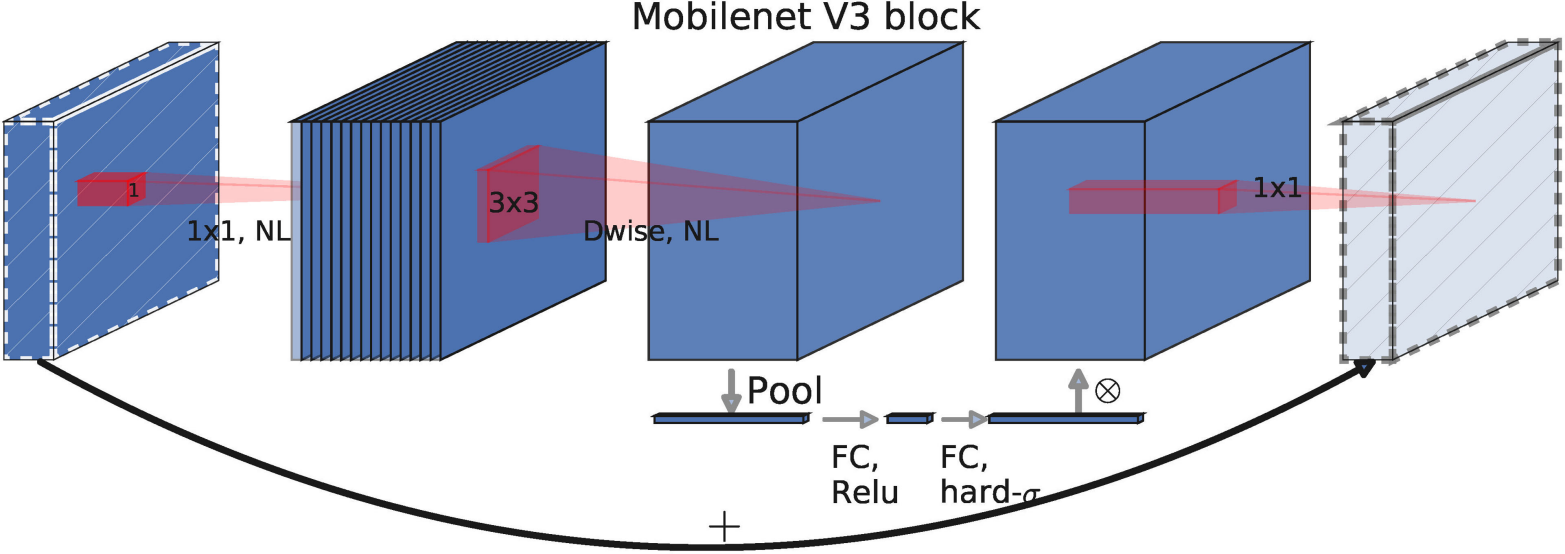
The architecture of the MobileNetV3s Basic Block. The basic block consists of four parts: depthwise convolution, linear bottleneck and inverse residual structure, and SE attention mechanism.

In this paper, the MobileNetV3s basic block is used, which can reduce the number of parameters and computations in the feature extraction process and achieve a good balance between speed and accuracy. As shown in **Figure 4**, the MobileNetV3s basic block is mainly composed of four modules: SE module, and DW convolution module (Zhang et al., 2020), combined with an inverted residual structure and a linear bottleneck structure.

##### 2.2.1.1 Depth-wise separable convolutions

Depth-wise separable convolution decomposes a standard convolution into a concatenation of two layers. First, it uses a lightweight depth-wise convolution layer ( Depthwise convolution ) to apply a single-channel convolution filter to each channel of the input feature map. Then, it connects a pointwise convolution layer ( Pointwise Convolution ), which generates a new feature map by linearly combining the features of each channel through a pointwise convolution operation.

The ratio of the total number of parameters between depth-wise separable convolution and normal convolution can be calculated as follows:


Y=X*f+b

The formula for traditional convolutional operation is shown in equation n, where * represents convolution operation. The output feature map dimension is $D_{F}\times D_{F}\times N$ , where N is the number of output channels, and the convolutional kernel dimension is $M\times D_{K}\times D_{K}\times N$ , where M is the number of input feature map channels. Therefore, the calculation of FLOPs for normal convolution is $N\times D_{F}\times D_{F}\times M\times D_{K}\times D_{K}$ .

Depth-wise separable convolution can be divided into two stages: depth-wise convolution and point-wise convolution. In the first stage, the input feature dimension of the depth-wise convolution is $D_{F}\times D_{F}\times M$ , the convolution kernel parameter is $D_{K}\times D_{K}\times 1\times M$ , and each channel corresponds to only one convolution kernel during convolution. The output dimension is $D_{F}\times D_{F}\times M$ , so the FLOPs of the depth-wise convolution is $D_{F}\times D_{F}\times M\times D_{K}\times D_{K}$ . In the second stage, the point-wise convolution has a convolution kernel parameter of 1×1×M×N and performs a 1×1 standard convolution on each feature, with an output dimension of $D_{F}\times D_{F}\times N$ . The FLOP calculation is $N\times D_{F}\times D_{F}\times M$ . Therefore, the parameter and FLOP ratio of the normal convolution and the depth-wise separable convolution can be obtained as


$$r_{parameters}=\frac{D_{K}\times D_{K}\times M+M\times N}{D_{K}\times D_{K}\times M\times N}=\frac{1}{N}+\frac{1}{D_{K}^{2}}$$

$$r_{FLOPs}=\frac{M\times D_{F}\times D_{F}\times D_{K}\times D_{K}+N\times D_{F}\times D_{F}\times M}{D_{F}\times D_{F}\times D_{K}\times D_{K}\times M\times N}=\frac{1}{N}+\frac{1}{D_{K}^{2}}$$

Where M is the number of input channels, $D_{F}$ is the size of the input feature map, $D_{K}$ is the size of the convolutional kernel, and N is the number of output channels.

The reduction of the parameter and FLOPs in depth-wise separable convolution can be approximately attributed to the parameter $D_{K}^{2}$ . When choosing k=3 , compared with the traditional convolution, depth-wise separable convolution reduces the parameter and FLOPs to 1/8−1/9 of the regular convolution while ensuring a slight drop in accuracy.

##### 2.2.1.2 Linear bottleneck and reverse residual structure

The reverse residual structure is as follows: The 1 × 1 convolution, also known as expansion convolution, expands the number of channels in the input feature map by a factor of “factor”, mapping the low-dimensional space to a high-dimensional space. Then, the 3 × 3 depth-wise separable convolution is used to greatly reduce the network’s parameter and computational complexity while ensuring the feature extraction capability and prediction accuracy of regular convolutions. Finally, the 1 × 1 convolution, combined with a linear activation function (Linear Bottleneck structure), is used to restore the number of output feature map channels to 1/factor. The use of a linear activation function can effectively reduce information loss during the transformation process from the high-dimensional to low-dimensional feature space. The input and output are only connected through the residual structure when they have the same number of channels. This structure reflects the compactness of the input and output, implements an internal nonlinear transformation to expand the features to a higher dimension, retains all the necessary information in the bottleneck, increases the feature expression ability, and reduces the network’s parameter count.

##### 2.2.1.3 SE module

Firstly, the channels of the input feature matrix are pooled to obtain an $R^{\text{channel}\times 1}$ -dimensional vector. Then, two fully connected layers are connected, with neuron numbers of channel and channel*1/4, respectively, and ReLu and h-swish activation functions are applied respectively. The SE module increases the weight of important parts of the results and reduces the weight of ineffective or less effective parts, thus training a better model.

In summary, compared with the CBS and C3 modules, the MobileNetV3s basic block has three advantages: 1) introducing depth-wise separable convolutions with lower computational cost to replace ordinary convolutions, thus reducing the number of parameters while maintaining network performance; 2) using linear bottlenecks and inverted residual structures to make more efficient layer structures by utilizing the low-rank properties of the problem; 3) using SE attention mechanism modules to increase the network’s sensitivity to effective information.

#### 2.2.2 DWC3-ghost

To further reduce model parameters and computational complexity for apple leaf detection on embedded devices, this paper proposes a new DWC3-ghost module using DWConv and GhostNet (Han et al., 2020) basic blocks, which is embedded in the neck network to replace the original C3 module, improving the efficiency of feature fusion in the model.

**Figure 5:**
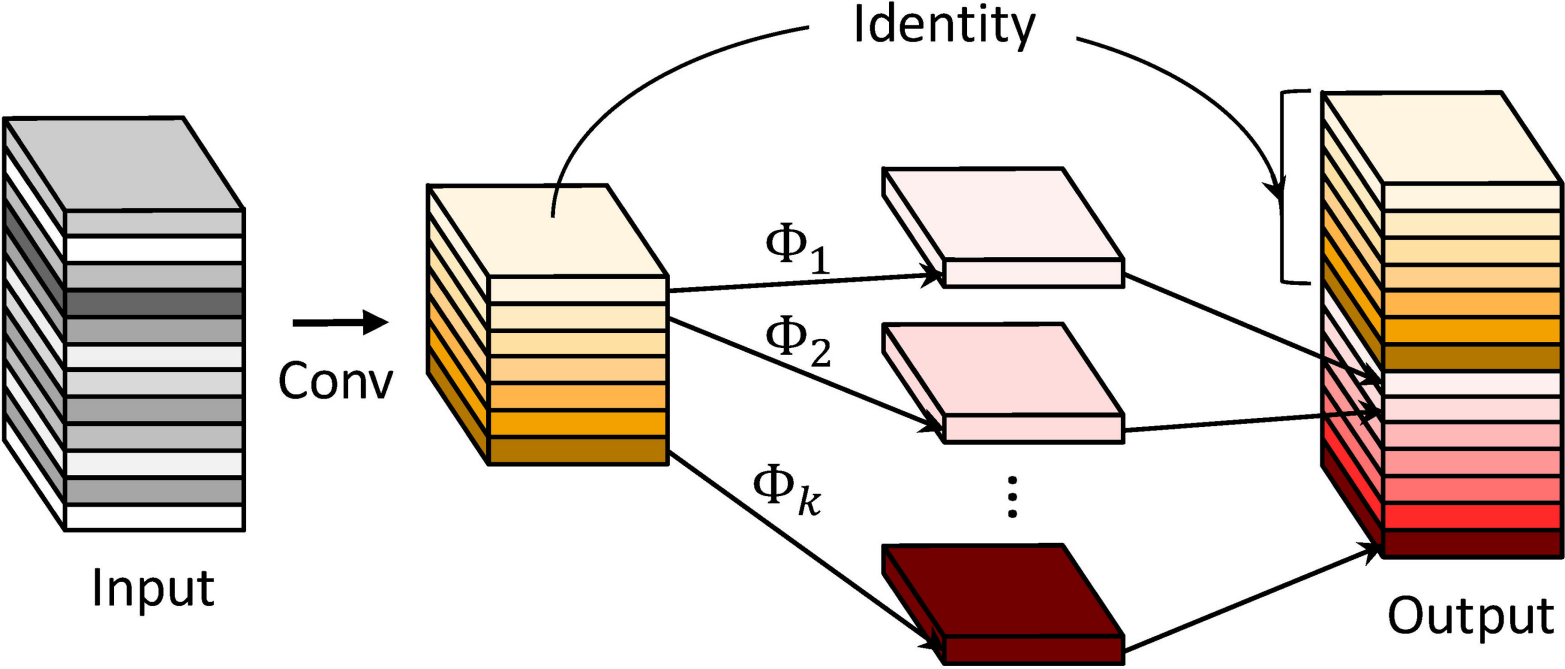
Ghost basic block computation: **(A)** general convolution with reduced number of channels. **(B)** Lightweight cheap linear transformation. **(C)** Stacking of the results of **(B)** computation.

As shown in **Figure 5**, in the ghost basic block, the input is first processed by a normal convolution (convolution + BN + activation function) to generate an intrinsic feature map with fewer channels, reducing the number of parameters. Then, the identity and inexpensive linear operation φ (only convolution) are used to enhance the features, generating the complete feature map.

In addition, lightweight operations such as low-channel convolution and inexpensive linear operations are used in the ghost module instead of traditional convolutional layers to generate redundant features, greatly reducing computational costs and achieving more efficient feature mapping and computation.

The FLOPs in the ghost module consist of two parts: the traditional convolutional layer that outputs a small number of feature maps and the lightweight and inexpensive linear transformation layer. The calculation process of the ghost module is shown in formula n:


$$Y^{`}=X*f^{`}$$

$$Y_{ghost}=\varphi_{j}\left(Y_{i}^{`}\right),j\in \left[1,s-1\right]$$

$$Y=Y^{`}+Y_{ghost}$$

The first stage outputs a small number of feature maps. The input feature map X has dimensions of $M\times D_{F}\times D_{F}$ , and the convolutional kernel has dimensions of $M\times D_{K}\times D_{K}\times N$ . The output feature map $Y^{`}$ has dimensions of $D_{F}\times D_{F}\times M$ . To simplify the calculation, the bias in the convolution calculation is omitted and the FLOPs are calculated as $D_{F}\times D_{F}\times D_{K}\times D_{K}\times M\times N$ . The second stage represents a lightweight and inexpensive linear transformation layer. $Y_{i}^{`}$ represents the i th feature map output from the first stage, $\varphi_{j}$ represents the j th ghost feature map generated by the j th linear operation, and $Y_{i}^{`}$ generates s−1 ghost feature maps. Finally, the ghost feature map $Y_{ghost}$ has dimensions of $D_{F}\times D_{F}\times N\times \left(s-1\right)$ .

The FLOP ratio of traditional convolutional layers and Ghost modules with the same number of channels and the same feature map size is shown in formula n. The Ghost module reduces the model FLOPs by around s times.


$$r_{s}=\frac{M\times D_{F}\times D_{F}\times N\times D_{K}\times D_{K}}{\frac{M}{s}\times D_{F}\times D_{F}\times N\times D_{K}\times D_{K}+\left(s-1\right)\times \frac{M}{s}\times D_{F}\times D_{F}\times d\times d}\approx s$$

where s represents the total mappings produced by each channel (1 intrinsic feature map and s-1 ghost feature maps) and d represents the average size of the convolutional kernel for linear operations.

**Figure 6:**
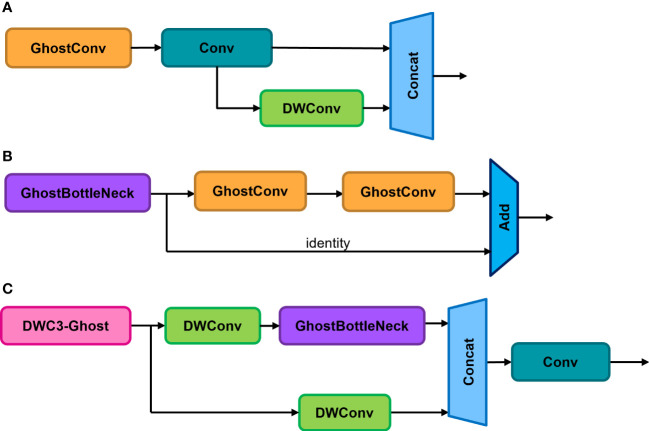
**(A)** Basic Ghost module. **(B)** Ghost-Bottleneck. **(C)** DWC3-Ghost.

Due to its simplicity and efficiency, the proposed GhostNet block was used to replace the Bottleneck in the original C3 convolution for lightweight implementation, as shown in **Figures 6A, B**. The CBS module was replaced by DWConv to design an efficient DWC3-ghost module, as shown in **Figure 6C**. The proposed DWC3-ghost module was embedded in the neck network to improve feature fusion efficiency. This can reduce the computational cost of the neck network while maintaining the expressive power of the features, thereby reducing the impact on detection accuracy.

#### 2.2.3 SPPCSCP_GC module

To make the model more lightweight and improve its real-time performance, this paper proposes using an improved SPPCSCP module (Wang et al., 2022). The SPPCSCP_GC replaces the original SPP module to further compress the model size, reduce the number of model parameters, and improve the efficiency of the model in the feature fusion stage.

The SPP module uses Maxpool on the feature map input into four branches with different scales, giving the model the ability to adapt to images of different resolutions. This effectively avoids image distortion caused by cropping or scaling operations on image regions and improves the scale-invariance of the image while effectively avoiding overfitting.

The CSP module halves the number of channels in the feature map and splits it into two branches, with one branch going through convolution processing and the other going through Bottleneck * N operations. The two branches are then concatenated, ensuring both accuracy and reduced computational cost.

**Figure 7:**
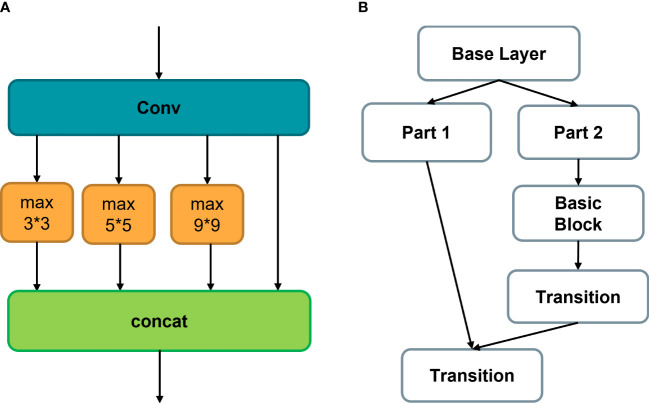
**(A)** Basic SPP module, **(B)** CSP structure, effectively reducing the number of model calculation parameters.

The proposed SPPCSPC module combines the SPP and CSP modules, as shown in **Figure 7**, with CSP as the main component. It retains the branch that performs a single convolution and adds an SPP module to the other branch. Finally, the two branches are concatenated to integrate all features, enhancing the effect of feature fusion while ensuring model lightweight.

**Figure 8:**
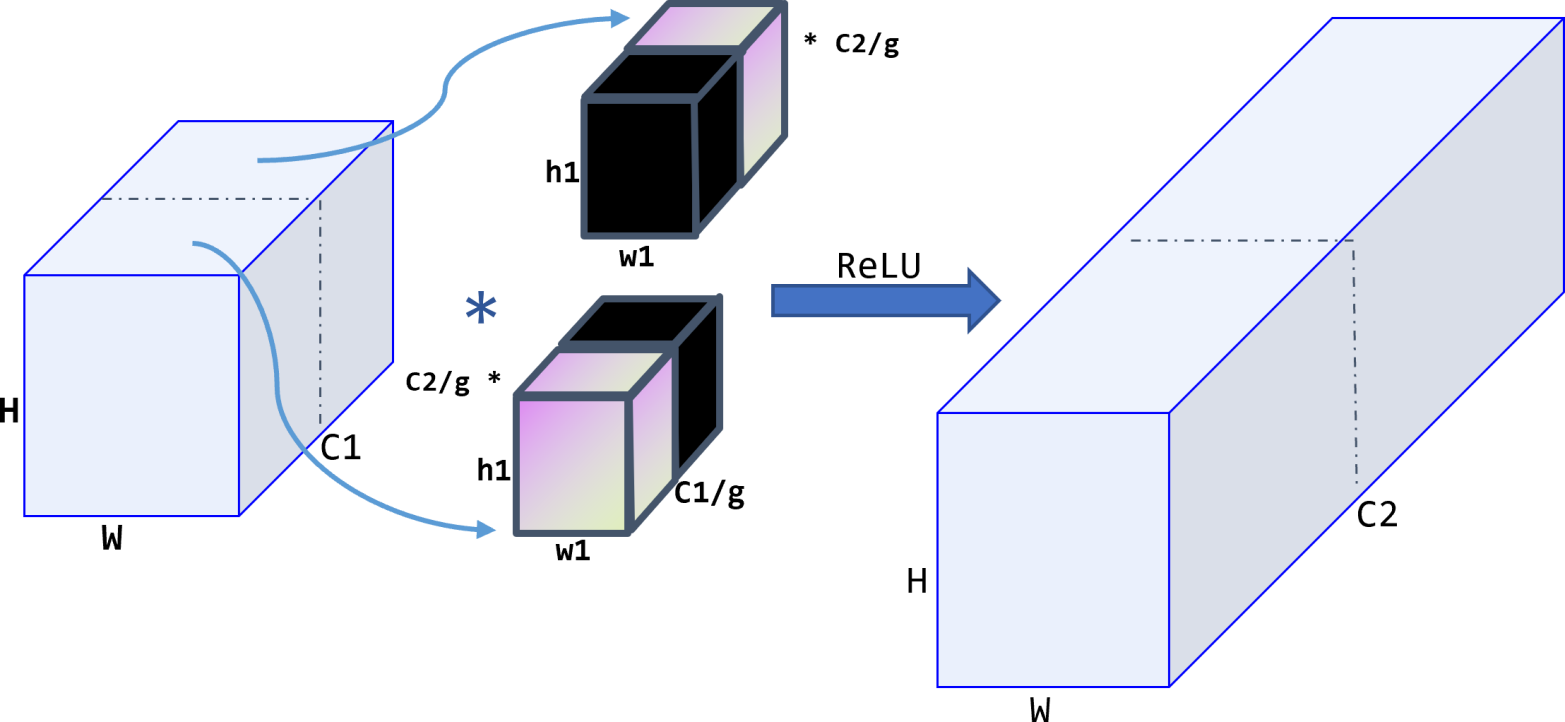
Group convolution operation procedure. The feature maps are processed in group, and each convolution kernel is divided into group accordingly.

To further improve the model’s computational speed, group convolution (Zhang et al., 2018) is applied to the SPPCSPC module in this model. Group convolution aims to process feature maps in groups, with each convolution kernel divided into groups and convolved within the corresponding group, as shown in **Figure 8**. The resulting feature maps are then concatenated together. Group convolution can increase the diagonal correlation between feature maps and significantly reduce training parameters, making it less prone to overfitting, similar to the effect of regularization.

Therefore, we propose an effective improved module SPPCSCP_GC, which combines SPP, CSP, and GC modules, so that the network structure can not only perform well under images of different resolutions but also effectively alleviate the problem of gradient vanishing. The module reduces the size of the model, ensuring inference speed and accuracy with fewer parameters and smaller FLOP values.

#### 2.2.4 Coordinate attention module

Improvements in the lightweighting of the neck network can make the model more lightweight and significantly improve its real-time performance, making it more convenient to deploy on mobile devices. However, these improvements inevitably lead to a decrease in detection accuracy. Therefore, we need to optimize the model’s performance on detection accuracy while not significantly affecting the computational cost.

**Figure 9:**
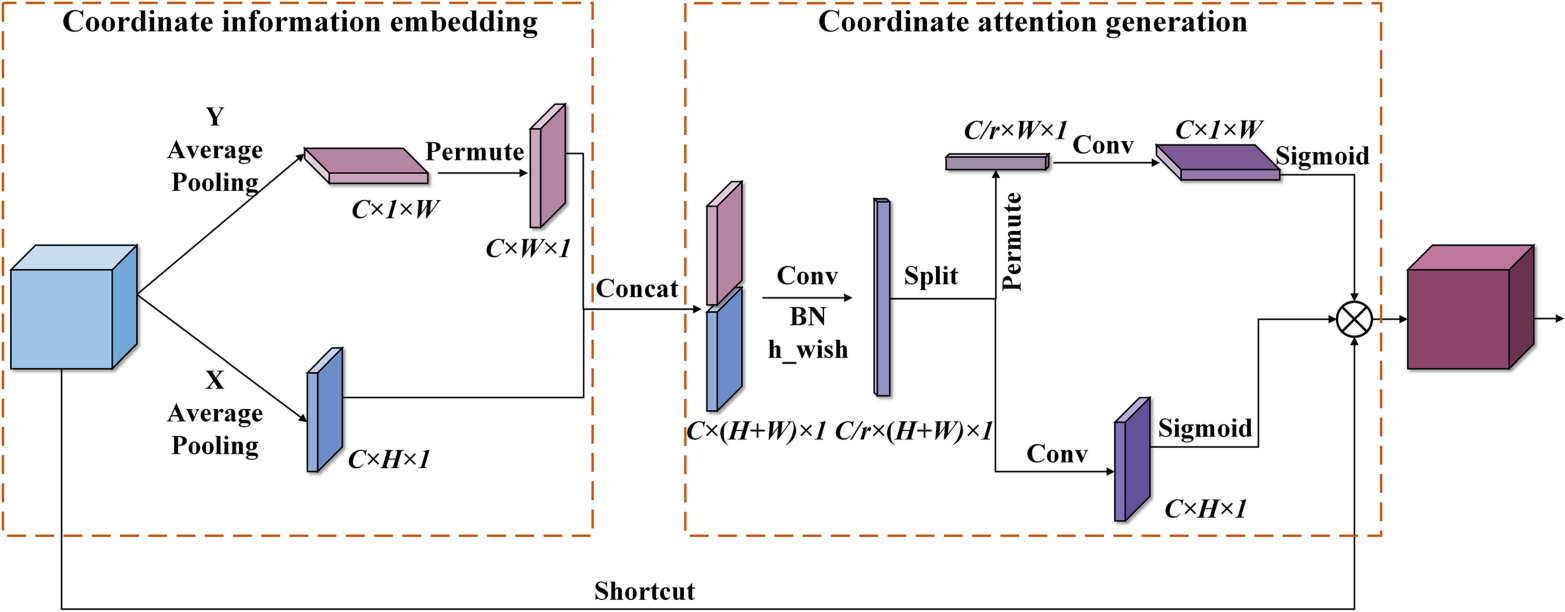
CA attention mechanism. Firstly, pool the input feature maps in the X(H) and Y(W) directions, respectively. Then, concatenate the output maps and use 1*1 convolution to reduce the dimension. Lastly, along the spatial dimension, perform the dimension raising operation and combine it with the sigmoid function to get the attention vector.

To sum up, we introduce the Coordinate Attention (CA) mechanism (Hou et al., 2021), as shown in **Figure 9**, which effectively addresses the issue of the SE attention mechanism’s focus only on building inter-channel dependencies and ignoring spatial features (Hu et al., 2018), as well as the CBAM attention mechanism’s introduction of large-scale convolution kernels for extracting spatial features but ignoring long-range dependencies (Woo et al., 2018). The CA module is flexible and lightweight enough to be easily incorporated into the core module of lightweight networks.

As shown in the above figure, the CA attention mechanism first performs pooling along the X and Y directions on the input feature map with size C×H×W , generating feature maps of size C×H×1 and C×1×W , respectively. Then, the two feature maps are concatenated to obtain a feature map of size C×(H+W) ×1 , which is then compressed from the C dimension to the C/r dimension using a 1×1  Conv, resulting in a feature map of size C/r×(H+W)*1 . Next, the h_wish function is used for non-linear activation, obtaining the intermediate feature representing the encoded information. The intermediate feature is then decomposed into a vertical attention tensor of size C/r×1×W and a horizontal attention tensor of size C/r ×H×1 , and each attention tensor is upsampled using a set of 1×1 Conv, increasing the number of channels from C/r to C. Finally, the sigmoid function is used for non-linear activation to produce the corresponding attention weights. The attention weights in the two directions are multiplied with the original feature map from the shortcut to obtain the attention-enhanced feature map.

The CA attention mechanism enables the network to collect information from a wider area rather than being biased toward a specific region, thus significantly improving detection accuracy. In addition, the CA module uses only 1×1 Conv kernels, two average pooling layers, and very few matrix transpositions, reducing the number of parameters and computational costs.

In this paper’s model, the CA module is embedded behind the intersection of all top-down and bottom-up information fusion in the neck network, effectively improving the detection accuracy loss caused by model compression. This allows the network to have attention to key information without incurring high computational costs, thus maintaining its efficiency and flexibility.

## 3 Results

In this section, the experimental setup, hyperparameter settings, and training strategies are detailed in Section 3.1. Then, Section 3.2 describes the evaluation metrics used to evaluate the performance of the model and their calculation formulas. Finally, in Section 3.3, the results of this article are explored in combination with an ablation experiment and visual analysis.

### 3.1 Implementation and setup

Experiments were conducted on the Featurize cloud server, with an Nvidia RTX 3090 graphics card configured for hardware, with 25.4 GB of graphics memory, and deployed on a Linux operating system. The implementation of the proposed method is based on Python 1.10.1.

The model training strategies are as follows. We set a dynamic learning rate to accelerate the network to the optimal value. The initial learning rate(lr0) was set to 0.1, and the OneCycleLR learning rate (lrf) is set to 0.01. The learning rate was updated every epoch until the final learning rate reached to lr0*lrf. The warmup_epochs was set to 3.0, with the warmup initial momentum set to 0.8 and the warmup initial bias lr set to 0.1. The training epoch was set to 500, and the batch size was set to 16. The weight attenuation was set to 0.0005, and the momentum was set to 0.937. The SGD optimizer is used to optimize the network parameters.

### 3.2 Evaluation metrics

To evaluate the detection precision of the proposed model ALAD-YOLO, we selected accuracy (P), recall rate (R), and average accuracy (mAP) as evaluation metrics. Among them, accuracy represents the ratio of positive samples correctly predicted to positive samples predicted by the model. It mainly measures the accuracy of the network in identifying positive samples. The recall rate represents the proportion of correctly identified positive samples to all positive samples, and it reflects the ability of the model in finding positive samples. mAP refers to the mean value of the average accuracies of all categories, which combines the detection performance between different categories. We calculated these evaluation metrics according to the following formulas:


$$P=\frac{TP}{TP+FP}$$

$$R=\frac{TP}{TP+FN}$$

$$AP=\int_{0}^{1}P\left(R\right)dR$$

$$mAP=\frac{\sum_{i=1}^{n}AP_{i}}{n}$$

where TP represents the number of positive samples correctly classified. TN is the number of correctly identified negative examples. FP represents the number of negative examples incorrectly classified as positive examples. FN represents the number of positive samples incorrectly classified as negative samples.

In addition to the above evaluation metrics to evaluate the detection performance of the model, we also use the number of parameters and FLOPs to evaluate the size and computational cost of the ALAD-YOLO model to select lightweight network to deploy on the mobile devices. Fewer parameters and FLOPs mean that under the same computing resources, the model can run more efficiently, while reducing memory usage and improving computing speed.

### 3.3 Ablation experiment and analysis

Ablation experiments are conducted to investigate the contribution of various modules in ALAD-YOLO, which improve the detection performance and reduce computational costs. The YOLOv5s model was used as the benchmark model. First, a lightweight network architecture is introduced to verify its impact on detection performance. Then, based on the lightweight network architecture selected, the improvement of network accuracy is verified by introducing modules, such as DWC3_Ghost module and CA module.

Firstly, different lightweight networks to improve the network performance are verified on the test set. The benchmark model YOLOv5s was compared with improved models, such as YOLOv5s GhostNet (Experiment 2) and YOLOv5s MobileNet (Experiment 3). Compared with the benchmark model YOLOv5s, the mAP50-95 in experiment 2 and experiment 3 decreased by 1.6% and 1.7%, respectively. However, in terms of parameter quantity, the FLOPs in experiment 2 and experiment 3 increased greatly by 49.4% and 54.4%, respectively. Specifically, the quantity decreased from 7 million to 3 million and 4 million. The results show that the backbone network composed of MobileNet basic blocks can significantly reduce the computing cost and the accuracy of mAP50-95 is only 0.1% lower than that of the Ghost module. Considering multiple factors, the experiment finally selects the backbone network composed of MobileNet basic blocks, which can significantly reduce the computing cost, and its impact on network accuracy is also acceptable.

The lightweight network structure can significantly reduce the size of the model and improve detection speed, with the cost of reducing the detection accuracy of the network. Therefore, some improved methods that can improve accuracy without introducing high computational costs are essential.

Secondly, based on the lightweight YOLO network composed of MobileNet basic blocks, the model performance changes caused by introducing different modules are verified. Experiment 6 introduces the SPPCSPC module to replace the original SPP module, better extracting and fusing feature maps. Experiment 7 uses the SPPCSPC_ GC module, which replaces the ordinary convolution in the SPPCSPC structure with group convolutions, effective feature fusion is ensured while achieving lightweight. Experiment 8 and experiment 9 respectively replace the C3 module in the neck part of the original network with DWC3 Host and DWC3 Faster structures. Based on experiment 9, experiment 10 and experiment 11 introduce an attention module to effectively extract key information about the detection results in the network. The improvements of the lightweight YOLO model on detection performance by introducing different improvement methods are shown in **Table 1**.

1. Experiment 6 and Experiment 7 show that using the SPPCSPC module and SPPCSPC_GC to replace the SPP module in the original network with the GC module can effectively improve the model detection accuracy, with improvements on the mAP50-95 by 3.0% and 6.4% on the test set, respectively. The addition of the SPPCSPC module in experiment 6 resulted in a significant increase of 5.2 G in the FLOPs of the model, which was somewhat outweighed by the 3% mAP increase. However, in experiment 7, after replacing the convolutions in the SPPCSPC module with group convolutions, SPPCSPC_GC was proposed. The mAP50-95 on the test set is increased by 6.4% with only a 0.9 G increase in FLOPs, significantly improving detection accuracy compared with the original benchmark network and meeting the lightweight requirements.
2. Experiments 8 and 9 show that using the DWC3-Ghost module and DWC3-Faster module instead of the C3 module in the original network neck section can effectively reduce parameter quantity and computational costs and has a certain improvement in detection accuracy. Compared with the benchmark network, experiment 8 and experiment 9 reduced FLOPs by 62.0% and 60.8%, respectively, and improved mAP50-95 on the test set by 0.1% and 0.5%, respectively. The results show that replacing the C3 module with a lightweight structure DWC3-Ghost can effectively compress the model size and computational cost and efficiently fuse and extract features to improve detection accuracy.
3. Experiments 9 and 11 show that adding a CA module to the lightweight YOLO network can effectively improve the detection accuracy of the network, increasing the mAP50-95 on the test set by 2.7%, while reducing the FLOPs by 0.1 G. Compared with the CBAM module added in experiment 10, its performance in the test set increased by 0.3% and the FLOPs also decreased by 0.1 G. Therefore, both have a slight increase in parameter quantity. The results show that compared with the CBAM module, embedding the CA module can better highlight information that is helpful for disease spot detection. Although it slightly increases the parameters of the model, it can well suppress useless information to improve the accuracy of the model, while reducing the FLOPs of the model.

**Table 1:** Comparison of experimental results based on the lightweight YOLOv5s model with different modules.

	Model	mAP50-95 (%)	mAP50 (%)	Parameters	FLOPs(G)
1	YOLOv5s	82.3	97.7	7,018,216	15.8
2	YOLOv5s-GhostNet	80.7	97.0	3,681,120	8.0
3	YOLOv5s-MobileNet	80.6	97.6	4,632,840	7.2
6	YOLOv5s-MobileNet-SPPCSPC	83.6	98.2	11,031,144	12.4
7	YOLOv5s-MobileNet-SPPCSPC_GC	87.0	98.4	5,665,768	8.1
8	YOLOv5s-MobileNet-SPPCSPC_GC-DWC3_Faster	87.1	98.7	4,640,616	6.0
9	YOLOv5s-MobileNet-SPPCSPC_GC-DWC3_Ghost	87.5	98.3	4,703,480	6.2
10	YOLOv5s-MobileNet-SPPCSPC_GC-DWC3_Ghost-CBAM	87.8	98.3	4,719,835	6.1
11	YOLOv5s-MobileNet-SPPCSPC_GC-DWC3_Ghost-CA	90.2	98.7	4,712,323	6.1

In summary, the proposed ALAD-YOLO model reduces the parameter quantity and FLOPs, significantly improving the speed of disease spot detection, whereas the impact on detection accuracy can be negligible. Therefore, the proposed ALAD-YOLO is more suitable for deployment on mobile devices with constrained resource and has the great detection performance required for practical applications.

On the test set with 2,748 images, the ALAD-YOLO network accurately identified three apple leaf diseases and healthy leaves, with mAP50 reaching 98.7% and mAP50-95 reaching 90.2%. The detection performance of each category is shown in **Table 2**.

**Table 2:** The detection performance of the ALAD-YOLO model on different categories of apple diseased leaves on the test set.

Class	Instances	P	R	mAP50	mAP50-95
All	3364	0.964	0.972	0.987	0.902
Mosaic	1788	0.961	0.971	0.985	0.894
Spot wilt	624	0.973	0.971	0.992	0.918
Leaf blight	952	0.958	0.974	0.985	0.892

**Figure 10:**
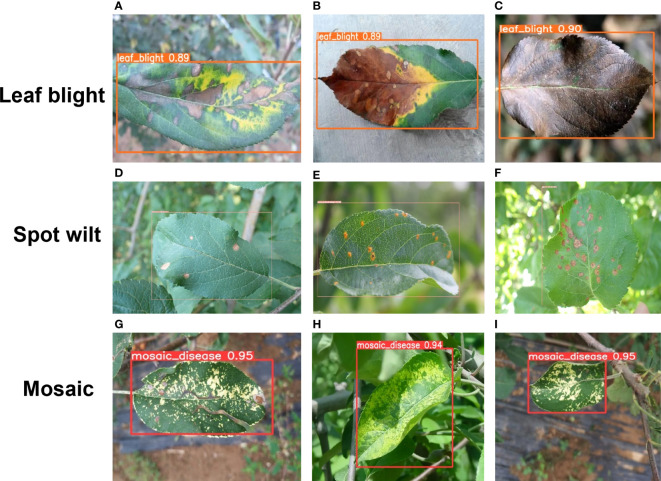
**(A–I)** indicate the detection effect with three samples randomly selected under three categories in the simple condition, i.e., a figure containing only one leaf to be detected. The bounding box shows the predicted label of each detected leaf and the confidence level of the prediction.

**Figure 11:**
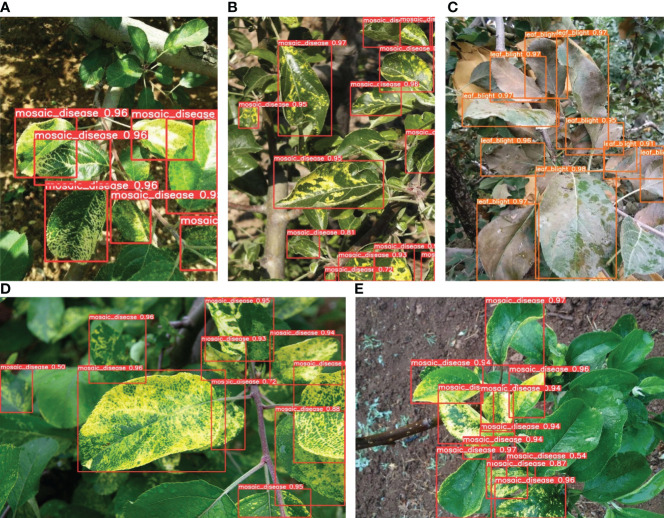
**(A–E)** indicate the complex cases, i.e., a figure containing multiple diseased leaves to be detected, and shading between the leaves is also common due to the high density.

**Figure 12:**
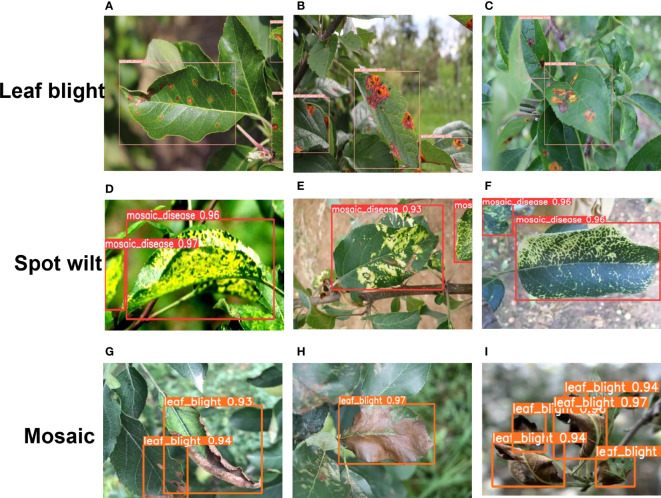
**(A–I)** indicate the detection effect in difficult cases, i.e., the leaves are at the edge of the figure or partially obscured. **(A, B, D–F)** show that ALAD-YOLO captures the edge information well. Also, **(C, G, I)** indicate that ALAD-YOLO has good detection ability for partially obscured diseased leaves.

To visualize the detection performance of the proposed method, **Figures 10**, **11** provide detection results of apple diseased leaf images in different scenarios. **Figure 10** visualizes the detection ability of the model in simple scenarios, where the simple environment refers to situations where the shooting is clear, the background is relatively simple and clear, the affected area of the apple is obvious, and the number of apple leaves included in the image is relatively small. From **Figures 10A–C**, it can be seen that our model can detect and judge three kinds of apple leaf disease accurately and simultaneously. Due to the proposed CA attention module, our model has a good ability to extract key information from images, resulting in high detection accuracy for different categories of apple leaf diseases. **Figure 12** indicates the detection effect in difficult cases, where the leaves are at the edge of the figure or partially obscured. From **Figures 12A, B, D–F**, it can be seen that our model can also accurately detect and judge the blades located in the edge region of the image. Also, **Figures 12C, G, I** indicate that our model can also accurately detect the leaves, which are partially obscured by others. At the same time, it can also grasp edge information in the image well.

To better demonstrate the advantages of the proposed model, **Figure 11** visualizes the detection capabilities of the model in complex scenarios. Due to the fact that most apple leaves are dense and have small spots in real environments, the detection of diseased leaves in dense environments is particularly important. However, our model is still very effective in this area. In the case of densely distributed apple leaves, the environment in which each apple leaf is located may not be the most suitable for testing. From **Figure 11**, it can be seen that areas with apple leaf diseases can be effectively detected, whether they are shaded areas, strong light exposure areas, shooting edge areas, or leaf stacking areas.

Our conclusion is that ALAD-YOLO can achieve the maximum mAP50-95 of 90.2% on the test set, which is 7.9% higher than the benchmark model YOLOv5s, while maintaining the minimum level of parameter quantity and FLOPs. Compared with other models, our model has the best recognition accuracy and achieves the fastest calculating speed and the smallest model size, meeting the requirements of real-time object detection for embedded mobile devices.

## 4 Discussion

Based on the YOLO-V5 architecture, we updated the original backbone network with Mobilenet-V3 and introduced several effective modules, proposing ALAD-YOLO to achieve both good detection accuracy and impressive speed. To further verify the model performance, we conducted many comparative experiments: compared with the regular YOLO-V5s model, ALAD-YOLO achieved a 7.9% improvement in accuracy while reducing FLOPs by 9.7G; for lightweight improvements such as YOLOv5s-GhostNet, YOLOv5s-MobileNet, and YOLOv5s-ShuffleNet, ALAD-YOLO showed a 10% or so increase in accuracy and 2–3-G improvement in speed. For networks with SPP, CA, CBAM, and other modules added, such as YOLOv5s-MobileNet-SPPCSPC and YOLOv5s-MobileNet-SPPCSPC_GC-DWC3_Faster, ALAD-YOLO was able to improve accuracy by around 3% while maintaining speed, achieving a balance between accuracy and lightweight and making it more suitable for completing tasks than other models.

In this study, we found some remaining issues in the model, which are common problems in current object detection models. In small object detection, such as detecting too many leaves, small spots, or unavoidable occlusion due to lighting, ALAD-YOLO may still have some missed detection or false detection (Alonso et al., 2020). We believe that more powerful and comprehensive data augmentation algorithms, such as random masking and noise introduction, can help the model learn more subtle features. Alternatively, taking pictures of leaves from multiple angles may also solve such problems.

We also studied the performance of ALAD-YOLO on mobile devices. In actual farms, data is usually collected through sensors, processed by edge computing devices, and then transmitted to cloud servers for more in-depth data analysis. Obviously, relying solely on smartphone-based applications cannot achieve round-the-clock disease detection on the farm. With the rapid development of artificial intelligence technology, suitable algorithms have been applied to the artificial intelligence of things (AIoT) (Chen et al., 2020). Our proposed ALAD-YOLO can be integrated into such a real-time observation system that provides farmers with environmental changes, so that disease can be judged more accurately and quickly. In addition, we also plan to compare our model with other SOTA methods on the Raspberry Pi platform to further evaluate its performance and provide more reference for future research.

At the same time, we hope that some high-performance edge computing modules or lightweight AI supercomputers can be applied to the field of agriculture. With such computing resources, the model will perform better than mobile platforms based on CPUs, and the corresponding models and algorithms will also have wider applications.

## 5 Conclusion

This paper proposes a lightweight apple leaf disease detection network, called ALAD-YOLO, to address the challenge of balancing accuracy and speed in the current detection of apple leaf diseases. Multiple data augmentation techniques are employed to enhance the apple leaf disease detection dataset for training and evaluation. ALAD-YOLO is an improved version of YOLO-V5s, with a more lightweight Mobilenet-V3 network as its backbone. This modification reduces the computational cost of feature extraction while ensuring accuracy. The proposed DWC3-ghost module is applied to the neck of the network, which improves the efficiency of feature fusion while maintaining its expressiveness. Moreover, the application of the SPPCSPC_GC module further enhances the model’s performance under different input resolutions. The introduction of the CA attention mechanism strengthens the model’s focus on the target, effectively compensating for the accuracy loss caused by previous lightweight operations. Experimental results show that ALAD-YOLO achieves a detection accuracy of 90.2% with 6.1 GFLOPs. Compared with existing models, ALAD-YOLO not only performs better in terms of accuracy but also has higher computational efficiency. Therefore, the proposed method provides excellent technical support for the real-time and accurate detection of apple leaf diseases. In the subsequent research, we will further optimize the performance of ALAD-YOLO in complex scenarios, so that it can have a wider range of applications.

## Data availability statement

The original contributions presented in the study are included in the article/supplementary material. Further inquiries can be directed to the corresponding author.

## Author contributions

WX and RW conceived and proposed the main approach. WX contributes to the collection and collation of data. WX conducted the experiment, and WX and RW worked together to analyze and improve the results. RW and WX wrote, revised, and reviewed the paper together. All authors contributed to the article and approved the submitted version.
